# Pilot-Scale Demonstration of Membrane-Based Nitrogen Recovery from Swine Manure

**DOI:** 10.3390/membranes10100270

**Published:** 2020-10-01

**Authors:** Beatriz Molinuevo-Salces, Berta Riaño, Matias B. Vanotti, David Hernández-González, María Cruz García-González

**Affiliations:** 1 Agricultural Technological Institute of Castilla y León, Ctra. Burgos, km 119, 47071 Valladolid, Spain; riairabe@itacyl.es (B.R.); hergonda@itacyl.es (D.H.-G.); gargonmi@itacyl.es (M.C.G.-G.); 2 Agricultural Research Service, Coastal Plains Soil, Water and Plant Research Center, United States Department of Agriculture, 2611 W. Lucas St., Florence, SC 29501, USA; matias.vanotti@usda.gov

**Keywords:** swine manure, gas-permeable membranes, ammonia recovery, nitrogen recycling

## Abstract

Gas-permeable membranes technology presents a high potential for nitrogen (N) recovery from wastewaters rich in ammonia (NH_3_). The EU project Ammonia Trapping (AT) is aimed at transferring knowledge from the lab-scale level to on-farm pilot-scale level, using this technology to recover NH_3_ from livestock wastewaters. The goal of this study is to report the results of an on-farm pilot-scale demonstration plant using gas-permeable membranes to recover N from raw swine manure. After a setup optimization of the plant, stable, and continuous operation was achieved. The maximum NH_3_ recovery rate obtained was 38.20 g NH_3_-N m^−2^ membrane day^−1^. This recovery rate was greatly affected by the temperature of the process. In addition to its contribution to NH_3_ emissions reduction, this technology contributes to the recovery of nutrients in the form of a concentrated stable ammonium sulphate solution. This solution contained 3.2% of N, which makes it suitable for fertigation. The economic approach revealed an economic feasibility of the technology, resulting in a cost of 2.07 € per kg N recovered.

## 1. Introduction

Reducing ammonia (NH_3_) emissions together with a lack of nutrient recycling are two major concerns in Europe. Agriculture is nowadays the largest source of NH_3_ emissions, which are related to diverse environmental problems and health risks in humans. More specifically, more than 91% of the total ammonia emissions in Spain in 2016 were related to agriculture [1]. Current regulation on air quality states a reduction commitment for NH_3_ annual emissions for each European country. In the case of Spain, this commitment accounts for a 3% reduction for any year from 2020 to 2029 and for a 16% reduction for years after 2030, compared to 2005, which is selected as the baseline year [2]. This commitment is forcing livestock farmers to adopt best available techniques (BAT) to abate these emissions [3]. Thus, the reduction of ammonia emissions has become not only an environmental but also an economic challenge for livestock farmers throughout Europe. 

There are different technologies for removing and recovering nitrogen from liquid livestock wastes, namely nitrification-denitrification, nitritation-denitritation, partial nitritation-Anammox, phototrophic systems, electrochemical cells, gas-permeable membranes, stripping, etc. [4,5]. Among the technologies that focus in NH_3_ recovery, gas-permeable membranes present several advantages since this process is carried out at low-pressure, they present a large contact area between the wastewater and the nitrogen trapping solution and the addition of alkali is avoided [6]. Ammonia passes through a microporous hydrophobic membrane by diffusion and an acidic trapping solution is used to recover it as a valuable (NH_4_)_2_SO_4_ solution. The driving force for ammonia transfer through the membrane is the difference in ammonia concentrations between the two phases, namely wastewater and acidic trapping solution. In ammonia recovery with gas-permeable membranes, the NH_3_ gas passes through the membrane and it is concentrated as a valuable fertilizer product.

This technology has been successfully applied to recover nitrogen from ammonia-rich wastewaters, such as livestock wastes or anaerobic digestates, producing valuable segregated fertilizer nutrients [6,7,8]. Diverse factors have been identified for improving the efficiency of this technology, namely the increase in the pH of the wastewater, the flow rate of the trapping solution, or the wastewater agitation [6,7,8,9,10]. For example, nitrogen recovery from raw swine manure reached 81% when pH in the wastewater was maintained in the range of 8.5–9. On the contrary, 55% of the initial nitrogen was recovered when no pH adjustment was performed [8]. 

Although there are many reported studies with this technology in the last few years, all of them have been carried out at laboratory scale. The EU project ammonia trapping (AT) was designed to transfer knowledge from the lab-scale level to on-farm pilot-scale level, in order to recover ammonia from livestock wastewaters by using the gas-permeable membrane technology. The main objective of the AT project was to reduce ammonia emissions from pig farms by capturing this nitrogen and producing a concentrated stable ammonium fertilizer. The target of the pilot AT project was a reduction in ammonia concentration in the livestock wastewater of more than 60%. The goal of this study was to report the main experimental results of an on-farm pilot-scale demonstration plant using gas-permeable membranes to recover nitrogen from raw swine manure. To the best of our knowledge, this is the first report of an on-farm pilot-scale plant using the gas-permeable membrane technology treating raw swine manure.

## 2. Materials and Methods 

### 2.1. Location of the Pilot Plant and Origin of Manure

**Figure 1:**
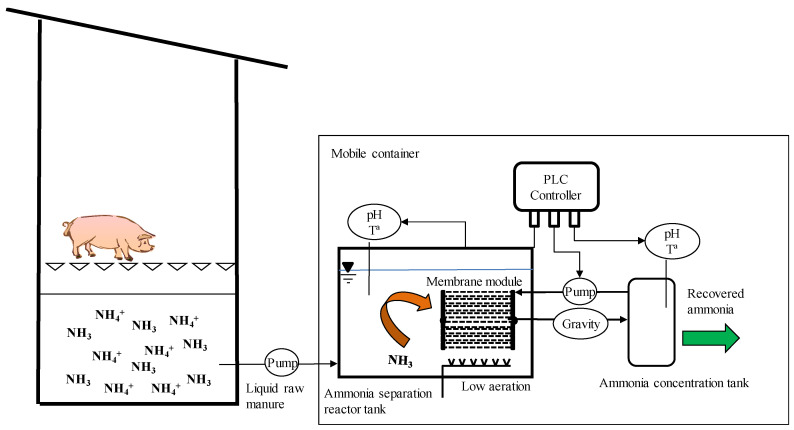
Scheme of the pilot plant.

**Figure 2:**
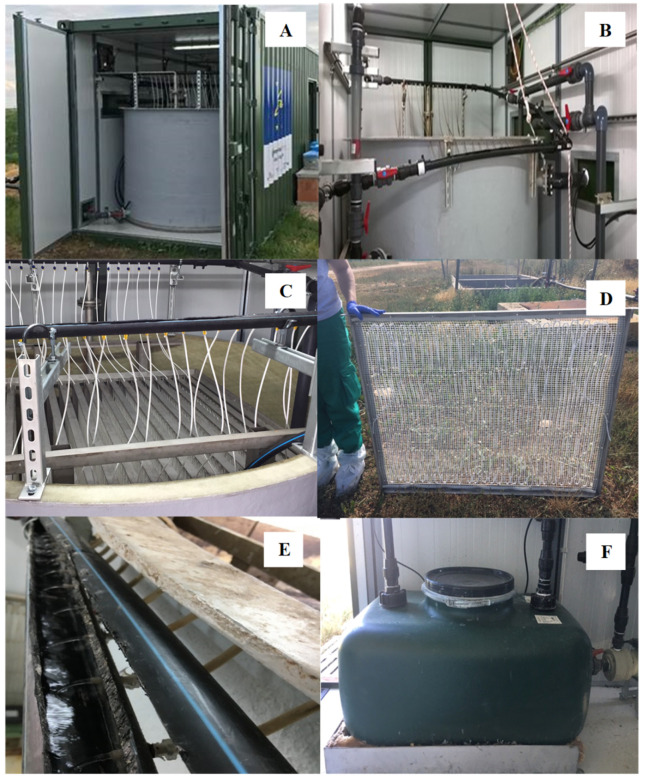
Pictures of the pilot plant. Shipping container, where the mobile pilot plant is located (**A**), manure tank with 16 membrane parallel panels (**B,C**), membrane panel (**D**), open pipeline for trapping solution collection (**E**), trapping solution tank (**F**).

Ammonia trapping experiments were carried out in a pilot-scale plant in Guardo (Palencia, Spain) (Figure 1). The pilot plant was located in a sow farm with 2800 animals, generating a volume of swine manure of approximately 17,136 m^3^ per year. More specifically, the pilot-scale plant was installed inside a mobile shipping container (Figure 2A), which was placed outside the farm building, next to a manure storage pit. Chemical characterization for raw swine manure is presented in Table 1.

### 2.2. Pilot Plant Configuration

**Figure 3:**
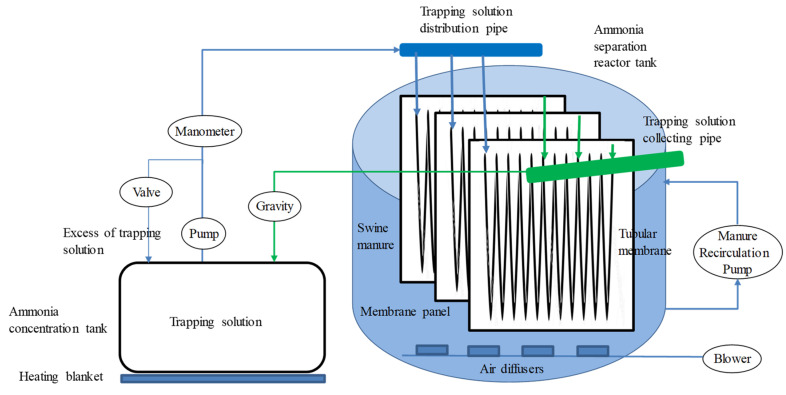
Schematic diagram of the pilot plant.

The pilot-scale plant was equipped as follows: A manure feeding pump, an ammonia separation reactor tank with a module of 16 membrane panels, a blowing air pump for aeration, a manure recirculation pump for mixing the reactor, a tank for ammonia concentration and for the trapping solution storage, a recirculation pump for the trapping solution, a heating blanket and a PLC control system (Figure 3). The feeding pump was submerged in a manure storage pit, feeding the reactor whenever necessary. The ammonia separation reactor tank had a total volume of approx. 5.85 m^3^ (diameter 2.15 m, height 1.61 m) and it contained a module with 16 parallel membrane panels placed in vertical configuration (Figure 2B,C; Figure 3). Each panel contained approx. 50 m of tubular gas-permeable membrane folded 25 times and held by plastic connections to a plastic support net (1 cm mesh) attached to a stainless-steel frame (1.30 m × 1.00 m) (Figure 2D). The membrane was made of expanded polytetrafluoroethylene (e-PTFE) material (ZEUS, Orangeburg, South Carolina, USA) with an outer diameter of 5.2 mm, a wall thickness of 0.64 mm, and a density of 0.95 g cm^−3^. The total surface of membrane was approx. 13.07 m^2^. The ratio of the tubular membrane length per manure volume was 0.16 m L^−1^ and the ratio of the membrane area per volume of manure was 0.0026 m^2^ L^−1^. Aeration in the reactor was provided by a blower (AIG, East London, South Africa, 0.75 kW, 1.67 m^3^ min^−1^) connected to seven air diffusers, which were placed in the bottom of the reactor [6]. Three air diffusers provided fine bubbles and four provided large bubbles. In addition to the mixing of the reactor liquid by the aeration, a pump (AIG, East London, South Africa, 1.5 kW, 50–400 L min^−1^) that recirculated the reactor liquid from the bottom to the top of the reactor also provided reactor mixing. 

The trapping solution (i.e., a solution of H_2_SO_4_ 1 N) was contained in a 0.25 m^3^ tank and it was continuously recirculated through the gas-permeable membranes by a recirculation pump (AIG, East London, South Africa, 0.56 kW, 50 L min^−1^). Specifically, the trapping solution was lifted to a distribution sealed pipeline connected to the 16 membrane parallel panels. Then, an open pipeline collected the trapping solution exiting the tubular membranes in the 16 panels (Figure 2C,E; Figure 3), which was returned to the concentrator tank by gravity (Figure 2F). It is noteworthy to mention that the pressure of this pump is a key issue in the durability of the membranes since a too high pressure could permanently damage the ePTFE membranes. However, low pressure prevented circulation of the trapping solution through all membrane panels. Therefore, circulation pressure of the trapping solution was particularly important to operate the pilot plant. The selected pressure to lift the trapping solution to the distribution sealed pipeline was 0.2 bar, being 0.5 bar the greatest outlet pressure for not damaging the membranes used. A heating blanket was placed below the ammonia concentration tank to reduce osmotic distillation of the membrane by increasing the temperature of the trapping solution [10]. 

A PLC system (Siemens, Munich, Germany) controlled and monitored the pilot-scale demonstration plant. More specifically, temperatures and the pH in the manure and trapping solution were continuously measured and recorded by the PLC. Aeration was used to keep up pH in the manure above 8.5 in order to increase available free ammonia to be recovered by the gas-permeable membranes. The PLC activated the air pump whenever the pH in the manure was below 8.5 and stopped the aeration at a pH of 8.5. The heating blanket was controlled also by the PLC with the objective of increasing the temperature in the acidic trapping solution. It was activated whenever the difference between the temperature in the manure and the trapping solution was below 2 °C.

### 2.3. Operating Procedures and Process Monitoring

The pilot-scale demonstration plant was evaluated during seven months, from April to November 2018. First, a setup period of 56 days was carried out to optimize the pilot plant operation (Section 3.1.1), after that, the pilot plant was operated in a batch mode. Five batch experiments (B1-B5) were carried out to evaluate the performance of the pilot plant at real environmental conditions, applying the same procedure for each batch run as follows. First, raw swine manure was pumped from the manure storage pit to the reactor, reaching a total working volume in the reactor of approx. 5 m^3^. The blower that aerated the manure was controlled by the PLC according to the pH of the raw swine manure. The manure was mixed by using a recirculation pump working in on/off cycles of 20/10 s. A volume of approx. 0.15 m^3^ of H_2_SO_4_ 1 N was used as trapping solution to concentrate total ammonia nitrogen (TAN) (Table 2). During the batch experiments, the acidic trapping solution was circulated without interruption through the membranes. In order to maintain the pH of the trapping solution below 2, a protocol was established. In this manner, concentrated H_2_SO_4_ (96%–98%, Panreac, Glenview, Illinois, USA) was manually added to the trapping solution to an endpoint of pH < 1 whenever the pH of the solution increased up to 2. This procedure was done under the supervision of the researchers. 

### 2.4. Sampling and Chemical Characterization

The pilot plant was located 3 h driving from the laboratory; therefore, researchers trained a farm worker to sample the manure and trapping solution every working day. These samples were kept at 4 °C and once a week they were transported in coolers with ice to the laboratory for analyses. Manure samples (100 mL) were taken from the reactor and trapping solution samples (10 mL) were taken from the nitrogen concentrator tank. TAN concentration and pH were determined in the laboratory. Hourly water temperatures were collected by the PLC and averaged during each batch period. Total alkalinity in the swine manure was determined at the beginning and at the end of each batch experiment. Analyses of total solid (TS), volatile solids (VS), total chemical oxygen demand (CODt), soluble chemical oxygen demand (CODs), total volatile fatty acids (TVFA), and total Kjeldahl nitrogen (TKN) were performed in swine manure at the beginning of each batch experiment. The pH was determined using a pH meter Crison Basic 20 (Crison Instruments S.A., Barcelona, Spain). Total alkalinity was determined by measuring the amount of standard sulfuric acid needed to bring the sample to pH of 4.5. Analyses of TS, VS, CODt, CODs, TAN, and TKN were performed in accordance with Standard Methods [11]. The concentration of TVFA (i.e., sum of acetic, propionic, butyric, iso-butyric, valeric, iso-valeric, hexanoic, and heptanoic acids) was determined using a gas chromatograph (Agilent 7890A) equipped with a Teknokroma TRB-FFAP column of 30 m length and 0.25 mm id followed by a flame ionization detector (FID). The carrier gas was helium (1 mL min^−1^). The temperature of the detector and the injector was 280 °C. The temperature of the oven was set at 100 °C for 4 min, then increased to 155 °C for 2 min, and thereafter increased to 210 °C. 

## 3. Results and Discussion

### 3.1. Pilot Plant Operation

#### 3.1.1. Problems Encountered in the Pilot Plant Start-Up

Prior to the batch experiments, there was an initial period of 56 days of operation in the pilot plant when diverse technical issues were solved. These issues were mainly related to the aeration, the corrosive effect of the trapping solution on the connectors, and the trapping solution recirculation. The initial aeration rate was not enough to increase the pH in the manure, therefore most of the TAN in the manure was in the form of NH_4_^+^ and not in the form of ammonia gas, resulting in low TAN recovery rates by the gas-permeable membrane on a membrane area basis (in the range of 2.6 and 6.0 g TAN m^−2^ day^−1^). The replacement of the initial blower (with a power of 0.4 kW and an airflow of 1100 L air min^−1^) for another with higher power and airflow (0.75 kW and 1666 L min^−1^, respectively) solved this issue. Second, the acidic trapping solution continuously recirculating in the lumen side of the membranes corroded the plastic connectors installed in the original pilot plant. This corrosion caused leakages of the trapping solution into the raw swine manure, leading to a low TAN recovery rate on a membrane area basis of 2.4 g TAN m^−2^ day^−1^. To solve this problem, the initial plastic connectors were replaced with acid-resistant connectors made of polypropylene. Third, the recirculation of the trapping solution through the lumen side of the membrane was set in discontinuous mode at the beginning, set in on/off cycles. This also resulted in low TAN recovery rates on a membrane area basis. The change to continuous recirculation of the trapping solution resulted in a better nitrogen mass transfer between the manure and the trapping solution, thus reaching TAN recovery rates up to 32.1 g TAN m^−2^ day^−1^. The changes performed during this initial period allowed an improvement on the operation of the pilot plant, thus preparing it for the batch experiments. More specifically, TAN recovery rates in the range of those targeted in the Ammonia Trapping project were reached during this initial period.

#### 3.1.2. Batch Experiments

Batch experiments were run with continuous recirculation of the trapping solution throughout the membranes. Table 1 shows the chemical composition of the swine manure used for the different batch experiments. In all the cases, it was a raw swine manure, with a high ratio VS/TS (0.70–0.78 g VS g TS^−1^) and a high concentration of TVFA (3.71–8.95 g COD L^−1^). The manure had TAN concentrations in the range of 2.30 to 3.05 g TAN L^−1^ and pH values were between 7.26 and 7.75. 

##### TAN Removal and Recovery: Effect of Temperature

**Figure 4:**
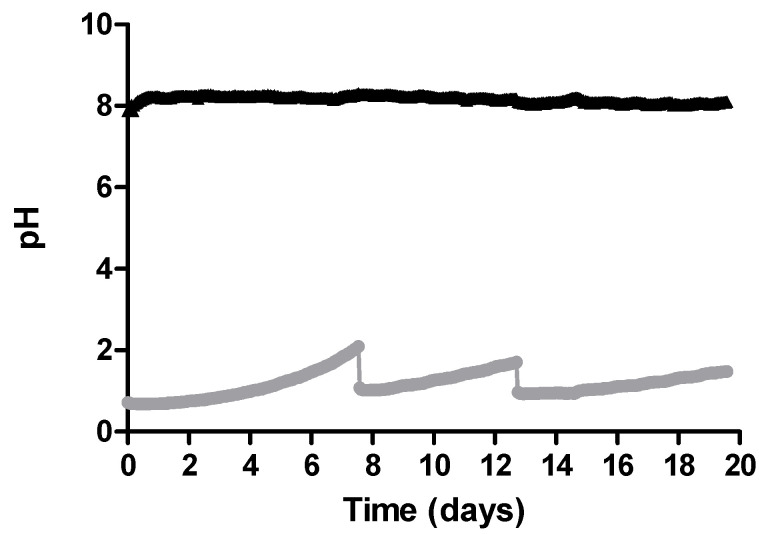
pH in the manure and in the acidic trapping solution during B3.

The provided aeration successfully increased pH in the manure, from averaged initial values of 7.60 ± 0.21 reaching averaged values of 8.61 ± 0.30 and remaining stable until the end of the experimental time (Table 3). The pH in the acidic trapping solution increased at some point due to ammonia reaction with the sulphuric acid. As soon as an increase in the pH was observed, it was reduced by adding concentrated sulphuric acid (Figure 4). 

The concentration of TAN in the manure was removed in the range of 14% to 49% (Table 3). Between 43% and 80% of the removed TAN was recovered as a (NH_4_)_2_SO_4_ solution (Table 4). The ammonia not recovered by the membranes was possibly stripped to the air, since no nitrate or nitrite was detected in the manure. The obtained removals and recoveries were lower than those in previous laboratory studies, probably due to the lower ratio of membrane area per volume of swine manure used in the pilot plant. More specifically, the pilot plant was operated with a ratio of 0.0026 m^2^ of membrane per liter of swine manure, while previous experiments at lab-scale were carried out with ratios 4–5 times higher (0.009–0.013 m^2^ L^−1^) [6,7,10,12,13]. The lower ratio of membrane in the pilot plant compared to previous studies implied a lower contact surface area between manure and trapping solution. Therefore, less ammonia gas passed the membrane per unit of time if compared to previous studies working with higher ratios of the membrane. For this reason, the removals and recoveries per unit of time obtained in the pilot plant were lower than those obtained in other studies. An increase in the ratio of membrane area per volume of manure is recommended to obtain higher nitrogen recoveries in the pilot plant. There is a variety of possible ways to increase the ratio, namely: (1) Increasing the number of membrane panels, (2) increasing the amount of membrane per panel, or (3) using a square tank, instead of the circular one, with the same size of the panels. Regarding membranes, it is worth mentioning that, even working with raw manure, no clogging problems were experienced after seven months of operation. 

**Figure 5:**
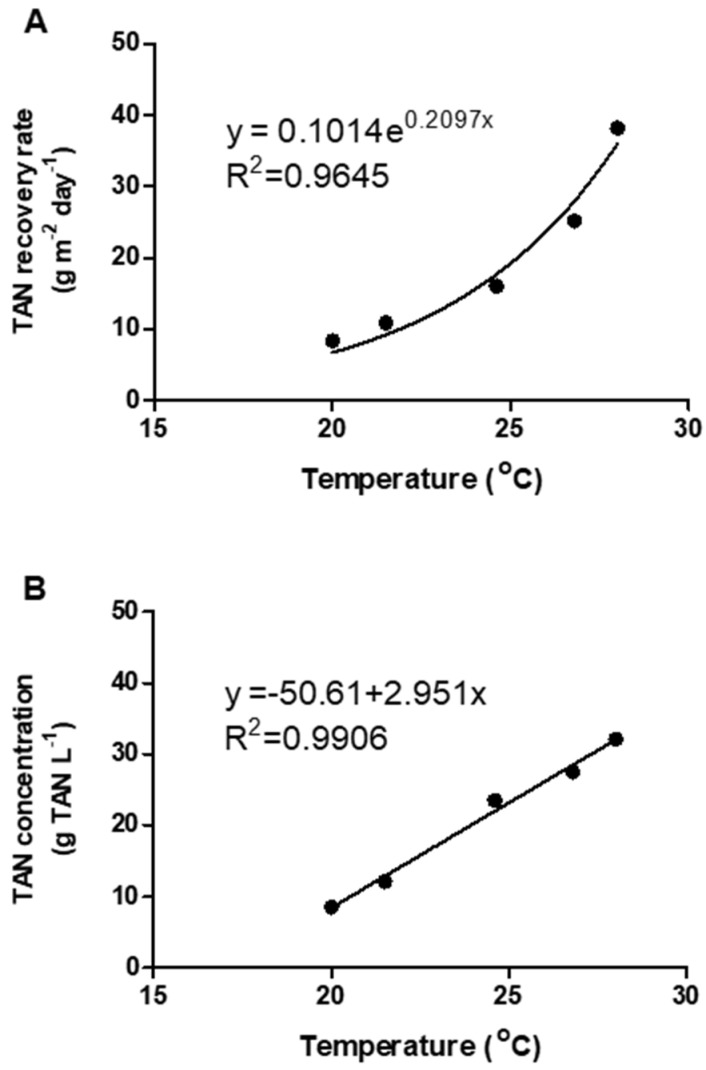
Relationships between (**A**) raw swine manure temperature and total ammonia nitrogen (TAN) recovery rate, and (**B**) raw swine manure temperature and TAN concentration in the acidic trapping solution.

The TAN recovery rates during the different batch experiments reached values in the range of 8.38 and 38.20 g TAN m^−2^ day^−1^ (Table 4). The average temperature in the liquid manure during each batch varied from 20 to 28 °C, corresponding to seasonal variations in air temperature (Table 2). B4 and B5, with temperatures in the manure lower than 25 °C, reached the lowest TAN recovery rates. Opposite to that, the conditions in B1, B2, and B3, with temperatures higher than 25 °C, resulted in high TAN recovery rates (Table 2 and Table 4). The TAN recovery rates calculated for the first seven days of operation followed the same trend (Table 4). A positive exponential relationship (R^2^ = 0.96) between temperature in the liquid manure and TAN recovery rate is shown in Figure 5A. Consequently, a high positive linear relationship (R^2^ = 0.99) was also observed between the temperature in the manure and the final TAN concentration in the trapping solution (Figure 5B). These results fit well with previous laboratory studies recovering TAN with gas-permeable membranes, which all were carried out at room temperature (22–25 °C). More specifically, these previous investigations reported TAN recovery rates in the range of 22.7 to 30.7 g TAN m^−2^ day^−1^ [6,10,12]. Therefore, it is recommended to maintain a temperature in the manure above 25 °C to optimize TAN recovery using gas-permeable membranes. The temperature inside the shipping container where the pilot plant was installed was dependent on seasonal variations so that two possible ways to increase temperature in the manure would be to insulate and heat the ammonia separation tank or to heat the entire room, (i.e., shipping container in this study). 

**Figure 6:**
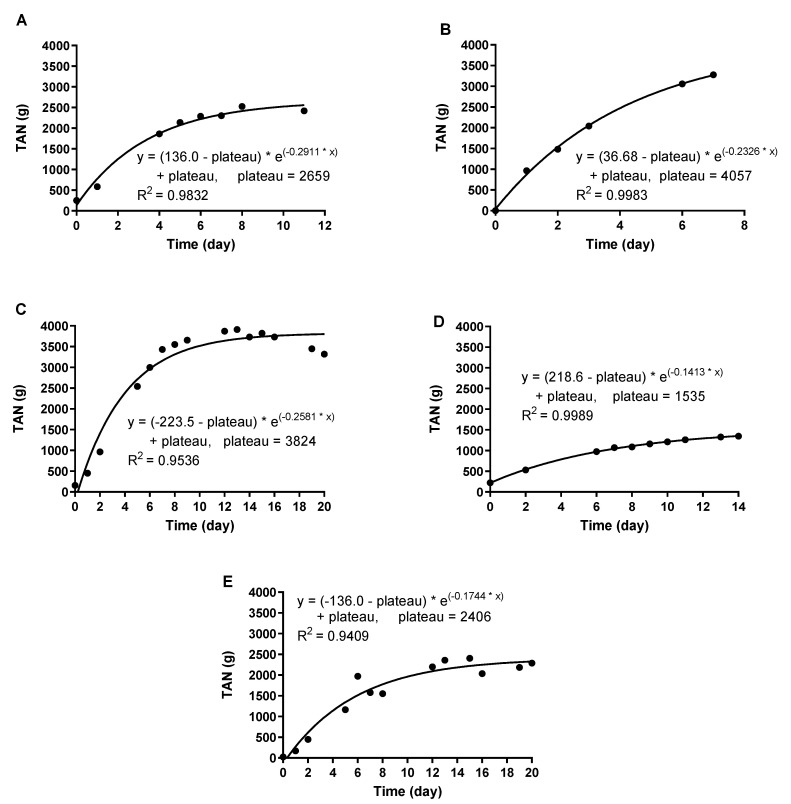
Mass of TAN in the trapping solution during B1 (**A**), B2 (**B**), B3 (**C**), B4 (**D**), and B5 (**E**).

Previous investigations using gas-permeable membranes to recover nitrogen at lab-scale in batch mode have demonstrated that the TAN recovery rate decreases with time during a batch, whereas it remains steady when operating at semi-continuous mode [10,12]. Since the pilot plant was operated in batch mode, the TAN recovery rate decreased with time, following a second-order curve (Figure 6), with most of the TAN recovered in the first week. 

The heating blanket installed in the pilot plant resulted in a successful reduction of osmotic membrane distillation (the passage of water vapor from the manure into the trapping solution). The occurrence of osmotic membrane distillation was only observed during B2 and B5, as it can be seen in the increase in the volume of the trapping solution (Table 2). These values were between 1.3 and 2.5 times lower than the value obtained by Riaño et al. [10] when working at lab scale in semi-continuous mode. Moreover, no change of color or turbidity was observed in the trapping solutions. Only minimal concentrations of minerals (0–20 mg of potassium, calcium, sodium, and iron L^−1^ day^−1^) and soluble organic matter (5–20 mg COD L^−1^ day^−1^) were found in the trapping solutions, which did not increase with time among the different batch experiments, indicating the good performance of the gas-permeable membranes at the end of the experimental time. 

During ammonia transfer through the membrane an accumulation of H^+^ occurs, thus alkalinity in the manure is consumed. A minimum initial alkalinity: Initial TAN ratio of 3.57 is needed to ensure a successful ammonia recovery by the membranes [14]. With lower ratios, all the alkalinity would be rapidly consumed, and the pH would decrease below seven, halting the N uptake by the gas-permeable membrane system [14]. The ratios for B1, B2, B4, and B5 were above that value, indicating that the manure tested had enough alkalinity for a successful N recovery by gas-permeable membranes at low-rate aeration. An exception was B3, where the initial ratio was 3.29 (Table 3). Alkalinity was reduced during experimental time approximately 33% ± 11% (range of 16%–41% depending on the batch experiment) but not exhausted, so that lack of alkalinity was not a barrier for N-recovery (Table 3).

##### Concentrated Ammonium Solution Obtained

The final product obtained after ammonia capture from the manure was a concentrated stable ammonium sulphate solution, with a maximum TAN concentration of 32.10 g TAN L^−1^ for an initial manure content of 2.82 g TAN L^−1^, which means a concentration 11 times higher than in manure (Table 3 and Table 4). The gas-permeable membrane technology has the capacity for segregating and concentrating ammonia from manure with diverse benefits for the farmers: (1) More control over nutrient application is possible, which avoids risks of N runoff or leaching, (2) reducing transportation costs of manure application, since the reduction of nitrogen concentration in the treated manure would permit to spread a higher amount of manure volume closer to the farms, (3) exporting the stable N solution off the farm, creating an additional income for the farmer, and (4) it would permit agricultural development to continuously increase while preserving the environment and the health of those living near livestock operations, thus improving general public relations.

With 3.2% of N (32.10 g TAN L^−1^), the ammonium sulphate solution obtained with the gas-permeable membranes process has a valuable market place for use in fertigation, whereby liquid nutrients in a stock mother solution are added to the irrigation water being supplied to crops [15,16]. Fertigation has diverse benefits such as fertilizer savings, better assimilation by plants, better distribution (both surface and in the soil profile), better nutrient loss control, great flexibility in the N application, and performance increase and quality improvement of the harvest [15].

### 3.2. Economic Approach

An economic approach for obtaining the annual cost of TAN recovery from raw swine manure with a pilot-scale plant using the gas-permeable membrane technology was carried out. The values used in these calculations are based on experimental data of this study together with the following conditions:

1. The annual production of raw swine manure in the farm is approx. 17136 m^3^ (i.e., 47 m^3^ per day), resulting from 2800 animals producing 6.12 m^3^ of manure per year each [17].
2. Raw swine manure contains an average of 2774 mg TAN L^−1^ (Table 1).
3. A TAN removal goal for the raw swine manure of approximately 90% is proposed.
4. TAN recovery efficiency using gas-permeable membrane is 62% and maximum TAN recovery rate of the pilot plant is 38.20 g TAN m^−2^ day^−1^ (Table 3 and Table 4).
5. Membrane cost is 115 € m^−2^ [6] and 10% of replacement per year is considered (this study).
6. Annualized costs of equipment are calculated using a 10-year useful life and 8% interest [6].
7. The amount of H_2_SO_4_ (98%) needed to capture TAN is 7.36 kg of acid per kg of N recovered (this study).

Taking into account the removal and the recovery percentages, 72 kg TAN per day (26,230 kg TAN year^−1^) should be recovered. With a maximum TAN recovery rate of 38.2 g TAN m^2^ day^−1^, a membrane surface of 1881 m^2^ is required to achieve this recovery. The cost of the membrane to start the pilot plant would be 216,341 €. Additional components, including feed pump, acidic solution recirculation pump, air blower, manure recirculation pump, tanks, heating blanket, and piping would account for approx. 23,007 € [6]. Thus, the annualized costs of equipment would account for 35,670 € per year. Moreover, the 10% considered membrane yearly replacing would result in 21,634 €. The annual cost of H_2_SO_4_ would be 55,985 € (0.29 € kg^−1^; [6]). Power consumptions for the different equipment are as follows: Blower 9 kWh d^−1^, feed pump 1.5 kWh d^−1^, acid recirculation pump 13.2 kWh d^−1^, and manure recirculation pump 36 kWh d^−1^. The total amount results in a total power consumption of 59.7 kWh d^−1^, resulting in an annual electrical cost of 2,822 € (unit cost in Spain = 0.1295 € kWh^−1^). Therefore, the estimated annual cost for a pilot plant using gas-permeable membranes in a swine farm with 2800 heads is around 116,111 € year^−1^. The ammonium sulfate potentially recovered per year (26,230 kg N) has an equivalent fertilizer value of 61,903 € assuming a value of 2.36 € per kg N as ammonium sulphate [6]. Therefore, the estimated net cost of the ammonia recovery per year is 54,208 €, or a cost of 2.07 € per kg N recovered. A summary of the capital costs, operational costs, and revenue is shown in Table 5. 

The energy consumption per kg of recovered nitrogen obtained in the present study was 0.68 kWh per kg recovered N. Compared to other technologies for nitrogen recovery, the energy consumption with gas-permeable membranes technology is much lower. For example, energy consumption to recover the ammonia by ammonia stripping technology has been reported to be 3.1 and 8.65 kWh per kg of recovered N [18,19]. These values using ammonia stripping are 5 to 13 times higher than the energy consumption obtained with the gas-permeable membranes. 

Although the economic approach reveals favorable results for this technology, the following aspects should be further investigated in order to improve the economy of the process:

1. Since the recovery of N is highly dependent on the temperature in the manure, a heating system for the winter months should be considered.
2. The evaluation of cheaper materials for the membranes and the use of recycled acids would be two examples of sustainable alternatives to reduce operational costs.

As mentioned before, best available techniques (BAT) can be adopted to abate NH_3_ emissions. In the case of the gas-permeable membrane technology, an important effort is being made towards the certification of this technology as a BAT to be used in swine farms in Europe.

## 4. Conclusions

This study showed that gas-permeable membrane technology was successful to recover nitrogen from raw swine manure in an on-farm pilot-scale plant. The maximum recovery rate per membrane area obtained was 38.20 g TAN m^−2^ day^−1^. The recovery rate was affected by the temperature in the raw manure in the range of 20 to 30 °C tested. Temperatures above 25 °C and ratios between membrane surface and manure volume higher than 0.01 m^2^ L manure^−1^ are recommended to optimize process performance. In addition to its contribution to NH_3_ emissions reduction, this technology recovers nitrogen in the form of a concentrated stable ammonium salt solution that can be used for fertigation. According to the experimental results, the net cost for this technology would be 2.07 € kg N recovered^−1^.

## Figures and Tables

**Table 1:** Chemical characterization for swine manure in the different batch experiments from B1 to B5. Average (SD) for duplicated analyses are shown.

Parameter	Unit	B1	B2	B3	B4	B5
pH		7.71	7.26	7.53	7.75	7.75
TS	g L^−1^	22.61 (0.36)	30.42 (2.23)	39.60 (0.79)	72.11 (3.09)	43.60 (0.35)
VS	g L^−1^	15.75 (0.40)	22.56 (1.99)	29.80 (0.83)	55.94 (2.85)	32.65 (0.29)
Ratio VS/TS		0.70	0.74	0.75	0.78	0.75
CODs	g L^−1^	15.12 (0.61)	13.74 (0.12)	13.84 (2.13)	19.42 (1.41)	10.86 (3.28)
CODt	g L^−1^	30.14 (1.93)	41.87 (3.24)	38.62 (1.36)	58.26 (1.08)	38.42 (1.68)
TVFA	g COD L^−1^	8.95 (0.19)	8.56 (0.04)	7.17 (0.28)	8.89 (0.00)	3.71 (0.07)
TAN	g L^−1^	2.79 (0.18)	2.82 (0.00)	3.05 (0.02)	2.92 (0.02)	2.30 (0.08)
TKN	g L^−1^	3.36 (0.00)	3.38 (0.01)	3.82 (0.00)	4.29 (0.01)	3.11 (0.02)

**Table 2:** Operational parameters during the pilot plant operation. Temperature values are averages (SD) for hourly data collected during each experimental batch.

Parameter	Unit	B1	B2	B3	B4	B5
Operation time	days	11	7	20	14	20
Trapping solution	m^3^	0.15–0.08	0.15–0.17	0.19–0.14	0.18–0.16	0.18–0.19
Manure	m^3^	4.86–4.68	4.97–4.72	4.94–4.74	5.12–5.06	5.10–5.06
Membrane surface	m^2^	13.07	12.03	13.07	10.60	13.07
Temperature manure	°C	26.8 (1.2)	28.0 (1.2)	24.6 (1.8)	20.0 (1.7)	21.5 (1.4)
Temperature trapping solution	°C	29.2 (2.0)	30.7 (1.3)	27.4 (1.7)	25.4 (2.3)	24.7 (1.8)

**Table 3:** Changes in chemical characteristics of raw swine manure during treatment in the pilot plant. SD are shown in parentheses.

Parameter	Unit		B1	B2	B3	B4	B5	Average B1–B5
pH	-	Initial	7.71	7.26	7.53	7.75	7.75	7.60 (0.21)
		Final	8.91	8.49	8.55	8.20	8.88	8.61 (0.30)
TAN	g L^−1^	Initial	2.79	2.82	3.05	2.92	2.30	2.78 (0.28)
		Final	1.99	1.92	1.54	2.51	1.22	1.84 (0.49)
Alkalinity	mg CaCO_3_ L^−1^	Initial	12794	11239	10033	13673	10126	11573 (1618)
		Final	7552	7937	5420	11345	6793	7809 (2198)
Removed TAN	%	-	28.53	31.87	49.46	14.30	46.82	34.20 (14.37)
Initial alkalinity: Initial TAN ratio	-	-	4.59	3.99	3.29	4.68	4.41	4.19 (0.57)
Alkalinity consumed	mg CaCO_3_ L^−1^	-	5242	3302	4613	2328	3333	3764 (1158)
Alkalinity consumed: TAN removed ratio	-	-	6.59	3.68	3.06	5.57	3.10	4.40 (1.60)

**Table 4:** Chemical characterization of the trapping solution during the pilot plant operation. SD are shown in parentheses.

Parameter	Unit		B1	B2	B3	B4	B5	Average B1–B5
TAN	g L^−1^	Initial	1.66	13.45	0.85	1.24	0.14	3.47 (5.61)
		Final	27.48	32.10	23.54	8.48	12.11	20.74 (10.09)
Recovered TAN in the trapping solution	%	-	79.69	66.23	42.81	59.32	62.10	62.03 (13.29)
TAN recovery rate	g TAN m^−2^ day^−1^	-	25.15	38.20	15.98	8.38	10.87	19.72 (12.16)
Initial seven-day TAN recovery rate	g TAN m^−2^ day^−1^	-	29.46	38.20	37.03	12.61	16.93	26.85 (11.62)

**Table 5:** Summary of capital costs and revenues of the pilot plant gas-permeable membrane system.

Capital Costs		
-	Initial Investment (€)	Annualized Costs: 8% Interest, 10-Year Life (€/Year)
Nitrogen Recovery Pilot Plant	239,348	35,670
Operational Costs		
**-**	-	Annual Costs (€/year)
Membranes Replacement (10%)	-	21,634
Chemicals (H_2_SO_4_)	-	55,985
Power (ƩkWh)	-	2822
Total operational annual costs-	-	80,441
Total Annualized Cost	116,111	
Revenue		
Sale of Fertilizer Products	(€/year)	
Recovered Nitrogen: 26,230 kg N/year (€ 2.36/kg N)	61,903	
Net Annual Cost	54,208	
